# Chemical and Bioactive Evaluation of Essential Oils from Edible and Aromatic Mediterranean Lamiaceae Plants

**DOI:** 10.3390/molecules29122827

**Published:** 2024-06-13

**Authors:** Rafael M. Spréa, Cristina Caleja, Tiane C. Finimundy, Ricardo C. Calhelha, Tânia C. S. P. Pires, Joana S. Amaral, Miguel A. Prieto, Isabel C. F. R. Ferreira, Eliana Pereira, Lillian Barros

**Affiliations:** 1 Centro de Investigação de Montanha (CIMO), Instituto Politécnico de Bragança, Campus de Santa Apolónia, 5300-253 Bragança, Portugal; rafael.sprea@ipb.pt (R.M.S.); ccaleja@ipb.pt (C.C.); tiane@ipb.pt (T.C.F.); calhelha@ipb.pt (R.C.C.); jamaral@ipb.pt (J.S.A.); iferreira@ipb.pt (I.C.F.R.F.); lillian@ipb.pt (L.B.); 2 Laboratório Associado para a Sustentabilidade e Tecnologia em Regiões de Montanha (SusTEC), Instituto Politécnico de Bragança, Campus de Santa Apolónia, 5300-253 Bragança, Portugal; 3 Department of Analytical Chemistry and Food Science, Instituto de Agroecoloxía e Alimentación (IAA)—CITEXVI, Universidade de Vigo, 36310 Vigo, Spain

**Keywords:** bioactive compounds, essential oil, Lamiaceae family, volatile compounds

## Abstract

The Lamiaceae family, which includes several well-known aromatic plants, is scientifically relevant due to its essential oils (EOs). In this work, four EOs from Mediterranean species, namely *Origanum vulgare* L., *Rosmarinus officinalis* L., *Salvia officinalis* L., and *Thymus vulgaris* L., were evaluated for their volatile profiles and the biological activity in vitro to assess their potential use in the food and cosmetic sector. GC/MS analysis revealed dominant compounds, such as carvacrol, thymol, and eucalyptol. Regarding biological action, the samples exhibited antioxidant, cytotoxic, anti-inflammatory, antimicrobial, and antifungal activities, with *O. vulgare* and *T. officinalis* standing out. *T. vulgaris* showed the lowest EC_50_ in the reducing power assay, and *O. vulgare* had the lowest EC_50_ in the DPPH assay. Most EOs also displayed excellent anti-inflammatory responses and antifungal properties, with *O. vulgare* and *T. vulgaris* also demonstrating antibacterial activity. All EOs from Mediterranean species showed cytotoxicity against tumoral cell lines. Overall, the selected EOs stood out for their interesting bioactivities, with the obtained results underscoring their potential as natural preservatives and bioactive agents in various industrial applications, including food, pharmaceuticals, and cosmetics.

## 1. Introduction

The Lamiaceae family comprises 236 genera and approximately 7200 plant species [1]. Throughout history, these plants have gained recognition for their culinary value as seasoning and flavoring agents, as well as for their traditional medicinal uses [2]. Scientific studies have reported the presence of compounds with significant relevance in different plant organs and essential oils within this family that are associated with their aromatic and bioactive properties [3]. Moreover, several members of the Lamiaceae family, such as mint, sage, oregano, thyme, basil, and rosemary, have been integral components of the Mediterranean diet, highlighting their extensive use in the culinary traditions of this region [4].

Essential oils (EOs) are complex mixtures mainly composed of several terpenes, commonly used as natural ingredients [5]. Essential oils of common spices, such as clove, cinnamon, basil, nutmeg, thyme, and oregano, are considered in the category Generally Recognized as Safe (GRAS) by the Food and Drug Administration (FDA). Furthermore, the GRAS status of EOs has engendered considerable interest in their use in the food preservation industry [6]. Contemporary trends in the food industry underscore a growing consumer preference toward environmentally sustainable options and a reduced reliance on synthetic chemical preservatives. In this context, EOs emerge as a promising alternative for replacing or decreasing the use of chemical additives, such as synthetic antioxidants and preservatives, and improving food packaging [7,8,9].

Some studies have highlighted the flavoring, antioxidant, and anti-inflammatory properties of different essential oils [10,11]. However, the application of EOs as food preservatives requires good knowledge of their properties, such as microbial and antioxidant sensitivity and the effects when in contact with the food matrix [12]. In addition to the food industry, EOs can also be used in sectors, such as cosmetics, to develop new fragrances and products for hair and skin care [13,14], and agriculture, as bioagents to prevent the development of pests during crop growth [15].

In this study, a preliminary selection of plants of this family cultivated in Portugal with potential for industrial-scale use was performed based on three main criteria, namely their status as aromatic plants commonly used in culinary practices, potential suitability for the extraction of EOs in accordance with the scientific literature, and origin as either native to the Mediterranean ecosystem or cultivated within this region. Consequently, four species of edible and aromatic plants, *Origanum vulgare* L. (oregano), *Rosmarinus officinalis* L. (rosemary), *Salvia officinalis* L. (sage), and *Thymus vulgaris* L. (thyme), were specifically chosen as focal subjects for essential-oil extraction by Clevenger hydrodistillation. Several studies have addressed certain parameters related to the composition and bioactivity of essential oils of said species; however, few articles have simultaneously evaluated the chemical composition and various biological activities of the same work. This article aims to fill this gap by providing a comprehensive and integrated analysis of the composition and antioxidant, cytotoxic, anti-inflammatory, and antimicrobial activities of essential oils extracted from Lamiaceae plants. Overall, the work seeks to contribute valuable insights for the future exploitation of aromatic plants at an industrial scale, potentially benefiting sectors such as food, pharmaceuticals, and cosmetics.

## 2. Results and Discussion

### 2.1. Composition in Volatile Compounds

GC/MS analysis allowed the identification of 90.3–92.3% of the compounds, considering all the EOs analyzed. In total, 9 compounds were identified in *O. vulgare* EO, 13 in *S. officinalis* EO, and 16 compounds in the EOs of *R. officinalis* and *T. vulgaris* (Table 1). All the essential oils showed a predominance of oxygenated monoterpenes, followed by monoterpene hydrocarbons. Nevertheless, *S. officinalis* and *O. vulgaris* presented a much higher content of oxygenated monoterpenes (81 and 86%, respectively) as compared to *R. officinalis* and *T. vulgaris* (53 and 48%, respectively), which in turn showed a higher number of monoterpenes (around 40%, while *S. officinalis* and *O. vulgaris* had <10%). In addition, *O. vulgaris* showed the presence of oxygenated sesquiterpenes, though in minor amounts (0.64 and 1.613%), while sesquiterpenes were also evidenced in *S. officinalis* EO (1.156%). However, *T. vulgaris* EO was the only sample that simultaneously presented these two groups, although sesquiterpenes were present as minority compounds (0.1%).

Carvacrol, a monoterpene recognized as having antioxidant, antimicrobial, and anti-inflammatory effects, as previously reviewed [16], was the main oxygenated monoterpene present in *O. vulgare* EO (85.78%), in agreement with previous studies reported in the literature. Caputo et al. comprehensively examined different drying methods of *O. vulgare* and reported carvacrol as the predominant compound in the EO obtained by the different drying techniques tested [17]. Elshafie et al. also reported carvacrol as the main constituent of *O. vulgare* EO [18]. Several beneficial effects have been associated with this compound, namely increased shelf-life of food products, mainly due to its powerful antioxidant and antimicrobial properties [19], as well as antimutagenic activity [18] and antimicrobial [20,21] and anticancer properties [22]. Carvacrol was also observed in the EO of *T. vulgaris*, although in smaller amounts (5.1%). In this species, the major terpene observed was thymol (41%), which is consistent with previous studies conducted by Ed-Dra et al., who investigated the use of *T. vulgaris* EO as a natural additive [23]. In this study, the authors also concluded that thymol is related to the inhibitory effect of *T. vulgaris* EO on different serotypes of Salmonella enterica, as also suggested by other works [24,25]. Thymol, an isomer of carvacrol, is directly associated with *Thymus vulgaris* as it is frequently found in this species [26]. This monoterpene has been significantly studied over the years, with various biological activities being attributed to it, notably antioxidant, anti-inflammatory, and antimicrobial properties [27,28,29]. Its applications, the identification of its mechanisms of action, and its pharmacokinetic studies position thymol as a potential agent for medicinal treatments [30].

Regarding *R. officinalis* EO, eucalyptol was the main terpene observed (34%). Eucalyptol is commonly found in Eucalyptus essential oil, but it can also be found in other plants. The literature reports this compound as an excellent antimicrobial; however, studies are still scarce [31]. Similar amounts of eucalyptol were also described by Amina et al. (37.97%), who suggested that the antioxidant activity exhibited by this oil can be related to the high content of this compound [32]. Moreover, the literature reports eucalyptol as a terpene that exhibits moderate inhibitory action against different microorganisms [33].

However, the EO of *S. officinalis* proved to be rich in camphor (29%), a terpene with antioxidant properties that can be used in food and pharmaceutical industries [34]. The extensive use of camphor in industries is due to the fact that this monoterpene is associated with the induction of apoptotic cell death through oxidative stress in a unicellular eukaryotic model [35]. Previous studies also found camphor as the major compound in the EO of this species, with its content ranging from 25.1 to 33.6% [36,37]. Khedher et al. concluded that the antioxidant and antimicrobial activities of the EO may be attributed to the composition of terpenes, suggesting that the presence of camphor contributes to its bioactive properties [37].

The significant presence of the aforementioned terpenes is consistently associated with biological activity. Characterizing and quantifying these compounds provide a better understanding of the EOs and their potential in future applications, including as natural additives in food products, antimicrobial agents, and antioxidants.

### 2.2. Antioxidant Activity

The results of the two in vitro assays (RP and DPPH) performed to evaluate the antioxidant activity are presented in Table 2. Overall, *O. vulgare* and *T. vulgaris* stood out for their better antioxidant properties as compared to the other samples, demonstrating the lowest values of DPPH and RP, respectively. *T. vulgaris* presented the lowest EC_50_ value in the reducing power assay (1.63 mg/mL), closely followed by *O. vulgare* (1.69 mg/mL), while *O. vulgare* performed better in the DPPH assay (9.23 mg/mL) than *T. vulgaris* (10.68 mg/mL). Laothaweerungsawat et al. reported that the EO of commercial *O. vulgare* from Mediterranean regions (Spain) exhibits a lower EC_50_ than the oil extracted from highland areas in tropical regions (Thailand); however, the results obtained in this work are in line with those reported in the literature [38]. Similar to *O. vulgare* EO, *T. vulgaris* EO also exhibited lower values of EC_50_, demonstrating good antioxidant activity, as previously reported by Mancini et al. when investigating the chemical composition and antioxidant potential of *T. vulgaris* collected in three different areas of Italy [39].

*R. officinalis* EO presented the highest value of EC_50_, particularly for DPPH (55.86 mg/mL), demonstrating its lower antioxidant capacity. While similar EC_50_ values were reported by el Kharraf et al. for the reducing power assay of the *R. officinalis* EO obtained by steam distillation [40], in previous studies conducted on the EO extracted through distillation from *R. officinalis* grown in Morocco [41], a lower EC_50_ value was obtained for the DPPH assay as compared to this study.

Regarding *S. officinalis* EO, the EC_50_ obtained in the DPPH assay is in agreement with previous results that reported similar values for the antioxidant activity (EC_50_ = 8.31 ± 0.55 mg/mL) [36]. However, better results were obtained for the reducing power assay as compared to those (EC_50_ = 28.5 ± 0.3) previously reported [37]. The variations in the antioxidant potential and the discrepancies in reported values for the same botanical species can be attributed to various factors, such as geographical region, cultivation conditions, and plant maturity [42]. The superior antioxidant response exhibited by the essential oils (EOs) from *Origanum vulgare* and *T. vulgaris* may be attributed to the presence of terpenes belonging to the phenol class, particularly carvacrol and thymol. Previous studies have demonstrated that terpenes belonging to the class of phenols exhibit higher antioxidant activity, followed by terpene aldehydes and ketones [43]. However, eucalyptol (ether) and camphor (ketone) were the predominant terpenes in *R. officinalis* and *S. officinalis*, with lower antioxidant activity being reported for their essential oils.

### 2.3. Cytotoxicity Potential

Cytotoxicity results of the EOs obtained from the different plants are presented in Table 3. Four tumoral cell lines, namely AGS (human gastric epithelial cell line), CaCo2 (human colorectal adenocarcinoma cell line), MCF7 (human breast carcinoma cell line), and NCI-H460 (human lung carcinoma cell line), were inhibited by all EOs with GI_50_ < 306 μg/mL, demonstrating their cytotoxic potential. The lowest GI_50_, denotating better activity, was observed for *O. vulgare* EO against all tumoral cell lines, ranging from 45 to 84 μg/mL. The cytotoxicity activity of *O. vulgare* EO rich in carvacrol was also reported against human skin cells [44]. On the contrary, the NCI-H460 cell line was the most resistant against the EOs tested as it showed the highest GI_50_ values. *R. officinalis* EO presented GI_50_ values from 60 to 306 μg/mL, which is in agreement with previous reports [45]. Likewise, *T. vulgaris* EO exhibited GI_50_ values close to those reported against three cellular lines, namely MCF7, HepG-2 (hepatic carcinoma), and HeLa [39] cell lines.

In addition, *S. officinalis* and *T. vulgaris* showed cytotoxic effects against the non-tumoral VERO cell line at a GI_50_ concentration of 243 μg/mL. Therefore, additional in vivo studies are necessary to verify the toxicity of these oils for specific applications.

### 2.4. Anti-Inflammatory Activity

All the EOs extracted from the four evaluated Lamiaceae species demonstrated excellent anti-inflammatory results (Table 4) based on the assay using the macrophage cell line RAW264., since all except *R. officinalis* presented better activity as compared to the positive control dexamethasone. Among the samples, *T. vulgaris* had the lowest GI_50_ (8 μg/mL), agreeing with the results previously reported [28,46], which also reported the capacity of *T. vulgaris* EO to inhibit NO production. *S. officinalis* and *O. vulgare* EOs also evidenced lower GI_50_ results as compared to the positive control. The EO obtained from *R. officinalis* oil showed a GI_50_ of 58.1 μg/mL, also in line with the literature [47]. The suppression of the inflammatory response of these EOs has also been reported in the literature [38,48,49].

### 2.5. Antimicrobial Activity

Table 5 reports the antibacterial and antifungal activity of the EOs against foodborne pathogens. None of the EOs showed bactericidal activity at the maximum concentration tested (2.5% *v*/*v*) against *P. aeruginosa*, *B. cereus*, and *L. monocytogenes*. Overall, *O. vulgare* EO demonstrated the highest antibacterial potency, followed by *T. vulgaris*. The former notably exhibited bacteriostatic activity against all tested strains, also evidencing the lowest MIC values across all bacteria. Additionally, it displayed bactericidal activity at low concentrations (0.08–0.63%) against four strains (*E. coli*, *S. enterica*, *Y. enterocolitica*, and *S. aureus*). *T. vulgare* EO was ineffective against *P. aeruginosa* but was able to successfully inhibit all other assayed bacteria. Compared to *O. vulgare* EO, *T. vulgare* EO exhibited a lower efficacy, as it generally presented higher MIC and MBC values for the same tested bacteria, except for *E. coli*, where it showed a lower MBC value.

Contrary to these two samples, *S. officinalis* and *R. officinalis* EOs showed low antibacterial activity, since for most bacteria, they only exhibited bacteriostatic activity at the highest concentration tested (2.5%, *v*/*v*) and bactericidal activity was only evidenced by *R. officinalis* EO against *E. coli*.

Previously reported results were consistent with those of this study, indicating that the EO of *O. vulgaris* could be a natural source of antimicrobial compounds. Using a different method, the agar disk diffusion method, Simirgiotis et al. reported a high sensitivity of bacteria against *O. vulgare* EO from Chile [50]. The EO was diluted in ethanol to an initial concentration of 10% *v*/*v* and tested against foodborne bacteria (*E. coli*, *P. aeruginosa*, *S. enterica*, *B. cereus*, and *S. aureus*), exhibiting MIC values between 0.08 and 0.63% (*v*/*v*) and MBC values from 0.08 to 1.25% (*v*/*v*). Kosakowska et al. also reported a significant inhibitory effect using the agar disk diffusion method for *O. vulgare* EO from Poland [51]. Regarding *T. vulgaris* EO, prior studies have also demonstrated its inhibitory and bacteriostatic effect using the agar disk diffusion method [24,52], aligning with the findings of this study.

Numerous studies have reported the fungicidal activity of EOs derived from Lamiaceae plants against a range of fungal pathogens, associating the activity to the volatile composition that can disrupt fungal cell membranes, inhibit fungal growth, and interfere with essential cellular processes [1,53,54,55]. In this work, all the tested EOs had good inhibitory and fungicidal potential as they showed values close to (*A. brasiliensis*) or even lower than (*A. fumigatus*) those of the positive control (ketaconazole). The EO of *O. vulgare* stood out for its low MIC value (0.08%, *v*/*v*) against *A. brasiliensis*. All tested EOs presented fungicidal values of 0.31%, with the exception of *R. officinalis* (0.63%). Notably, *A. fumigatus* showed greater sensitivity to all tested EOs, with MIC = 0.08% and MBC = 0.31%.

## 3. Materials and Methods

### 3.1. Sample Preparation

*Origanum vulgare* L., *Rosmarinus officinalis* L., *Salvia officinalis* L., and *Thymus vulgaris* L. were kindly provided in dried form by the certified industry Deifil (Póvoa de Lanhoso, Portugal). Aerial parts of the four Lamiaceae plants were transported to the laboratory facilities and stored in a dry place, protected from light and moisture. Before analysis, they were ground to a powder (model A327R1, Moulinex, Barcelona, Spain) to approximately 20 mesh and stored at room temperature.

### 3.2. Volatile Compounds

The aerial parts of the aromatic edible plants were submitted for essential-oil extraction by hydrodistillation. For this purpose, the plant parts were kept in the Clevenger apparatus for 3 h with distilled water in a ratio of 1:20 (*m*/*v*). The EO was recovered without the addition of any solvent, and anhydrous sodium sulfate was added to eliminate any traces of water from the samples. Subsequently, the EO was diluted in n-hexane (1:100) and analyzed by GC/MS using a Perkin Elmer system with a Clarus^®^ 580 GC module, Perkin Elmer, Waltham, MA, USA and a Clarus^®^ SQ 8 S MS module, equipped with a DB-5MS fused-silica column (30 m × 0.25 mm i.d., film thickness 0.25 μm; J & W Scientific, Inc., Folsom, CA, USA), under the conditions previously described by [56].

### 3.3. Bioactivity Evaluation

#### 3.3.1. Antioxidant Activity

Antioxidant properties were evaluated by two in vitro assays, reducing power (RP) and 2,2-diphenyl-1-picrylhydrazyl (DPPH), as previously described [56]. Results were expressed in EC_50_ for the DPPH assay, which translates the concentration of the EO that scavenges 50% of the radicals, and in EC_50_ for RP, corresponding to 0.5 of absorbance at 690 nm. For both methodologies, a SpectroStar nano-spectrophotometer reader (Labtech, Ortenberg, Germany) was used. Butylated hydroxytoluene (BHT), calcium ascorbate (E302), and sodium metabisulphite (E223), as organic molecules commonly used as food additives, were used as positive controls.

#### 3.3.2. Cytotoxicity Activity

A sulforhodamine B colorimetric assay was performed to evaluate the cytotoxicity potential of the samples against four human tumor cell lines, namely AGS (human gastric epithelial cell line), CaCo2 (human colorectal adenocarcinoma cell line), MCF7 (human breast carcinoma cell line), and NCI-H460 (human lung carcinoma cell line), following the methodology previously described by [56]. Furthermore, cytotoxicity against non-tumoral cells was assessed in VERO (African green monkey kidney) cells. All cell lines used in the in vitro assays were obtained from Leibniz-Institute DSMZ-German Collection of Microorganisms and Cell Cultures GmbH. Ellipticine was used as the positive control. Results were expressed as the concentration of essential oil with the ability to inhibit 50% of cell growth (GI_50_).

#### 3.3.3. Anti-Inflammatory Activity

The murine macrophage cell line (RAW 264.7, European Collection of Authenticated Cell Cultures) was used to evaluate the anti-inflammatory potential, as described by [56]. Furthermore, a commercial corticosteroid (dexamethasone) was used as a positive control. The results were presented as the concentration of extract causing 50% inhibition of nitric oxide (NO) production (IC_50_, µg/mL).

#### 3.3.4. Antimicrobial Activity

The antibacterial activity was evaluated using three Gram-positive bacteria, namely *Bacillus cereus* (ATCC 11778), *Listeria monocytogenes* (ATCC 19111), and *Staphylococcus aureus* (ATCC 25923), and four Gram-negative strains, namely *Escherichia coli* (ATCC 25922), *Pseudomonas aeruginosa* (ATCC 9027), *Salmonella enterica* (ATCC 13076), and *Yersinia enterocolitica* (ATCC 8610), following the protocol described by [57]. Two micromycetes, *Aspergillus fumigatus* (ATCC 204305) and *Aspergillus brasiliensis* (ATCC 16404), were used to determine the inhibitory and fungicidal activity of the essential oils, as described by [56]. Results were expressed as the minimum inhibitory concentration (MIC) and the minimum bactericidal or fungicidal concentration (MBC/MFC), as % (*v*/*v*) of essential oil.

### 3.4. Statistical Analyses

Results of assays performed in triplicate were presented as the mean ± standard deviation. The statistical software used for data analysis in this study was SPSS Statistics (IBM SPSS Statistics for Windows, version 23.0). To assess the statistical differences among multiple groups, analysis of variance (ANOVA) was performed, followed by a post hoc Tukey test. The threshold for statistical significance was set at *p* < 0.05. In cases where the sample size was less than three, Student’s *t*-test was used to evaluate significant differences between two samples, with a significance level of *p* = 0.05.

## 4. Conclusions

This study provides valuable scientific knowledge about the composition and bioactivity of EOs extracted from four Lamiaceae plants. The EOs from the selected plants exhibit a distinct composition, although all are dominated by oxygenated monoterpenes, with carvacrol being the main compound in *O. vulgare* EO, thymol in *T. vulgaris* EO, eucalyptol in *R. officinalis* EO, and camphor in *S. officinalis* EO. The presence of these bioactive compounds aligns with the previous literature and supports the diverse and significant biological activities of the four EOs, including antioxidant, cytotoxic, anti-inflammatory, and antimicrobial properties. Overall, *O. vulgare* and *T. vulgaris* EO demonstrate particularly noteworthy antioxidant and antimicrobial properties, with *O. vulgare* EO also exhibiting remarkable cytotoxic activity against various tumoral cell lines. These findings support their potential use in various applications, including in food preservation, as antioxidant ingredients, in applications in smart packaging and antimicrobial packaging, in the cosmetic and pharmaceutical fields as natural preservatives, and in aromatherapy applications. In the future, essential oils can be subjected to the HET-CAM assay to assess the biocompatibility of the compounds, thereby expanding future prospects for the safe and effective use of these products in various applications.

## Figures and Tables

**Table 1:** Chemical volatile profiles of the four Lamiaceae essential oils extracted by hydrodistillation by the Clevenger apparatus.

*Origanum vulgare* L.					
Number	Compound	RT (min)	LRI ^a^	LRI ^b^	Relative % ^c^
1	α-Pinene	8.458	926	932	0.076 ± 0.001
2	Camphene	9.123	940	946	0.048 ± 0.003
3	β-Pinene	10.138	985	974	0.012 ± 0.001
4	*o*-Cymene	12.798	1018	1022	0.53 ± 0.02
5	γ-Terpinene	14.461	1052	1054	0.82 ± 0.03
6	Terpinolene	16.876	1102	1088	4.4 ± 0.2
7	Thymol	25.61	1287	1289	0.013 ± 0.0001
8	Carvacrol	26.975	1318	1298	85.78 ± 0.02
9	Caryophyllene oxide	37.336	1568	1582	0.64 ± 0.04
	Total identified (%)				92.3 ± 0.2
	Monoterpenes				5.9 ± 0.3
	Oxygenated monoterpenes				85.8 ± 0.02
	Oxygenated sesquiterpenes				0.64 ± 0.04
	Not identified				7.7 ± 0.2
*Rosmarinus officinalis* L.					
Number	Compound	RT (min)	LRI ^a^	LRI ^b^	Relative % ^c^
1	Santolina triene	7.95	915	908	0.1 ± 0.01
2	α-Pinene	8.69	930	932	24.1 ± 0.2
3	Camphene	9.26	943	946	3.95 ± 0.01
4	Dehydrosabinene	9.4	946	956	0.17 ± 0.01
5	β-Pinene	10.42	968	974	0.32 ± 0.01
6	β-Myrcene	11.4	989	991	8.1 ± 0.3
7	*o*-Cymene	12.83	1019	1022	1.28 ± 0.01
8	ρ-Cymene	12.99	1022	1023	0.6 ± 0.001
9	Eucalyptol	13.39	1030	1031	34 ± 1
10	γ-Terpinene	14.46	1052	1060	0.49 ± 0.01
11	Terpinolene	15.7	1078	1088	0.21 ± 0.01
12	Camphor	18.75	1141	1142	5.9 ± 0.05
13	α-Terpineol	21.41	1195	1189	2.15 ± 0.01
14	Verbenone	21.81	1204	1204	9.4 ± 0.1
15	Bornyl acetate	25.14	1277	1284	1.23 ± 0.01
16	Methyleugenol	30.37	1396	1402	0.22 ± 0.01
	Total identified (%)				91.9 ± 0.1
	Monoterpenes				39.2 ± 0.6
	Oxygenated monoterpenes				52.7 ± 1.1
	Not identified				8.1 ± 0.1
*Salvia officinalis* L.					
Number	Compound	RT (min)	LRI ^a^	LRI ^b^	Relative % ^c^
1	α-Pinene	8.475	926	932	3.3 ± 0.1
2	Camphene	9.175	941	946	4.4 ± 0.5
3	β-Pinene	10.383	967	974	0.79 ± 0.02
4	β-Myrcene	11.188	984	991	0.53 ± 0.04
5	Eucalyptol	13.253	1027	1031	17 ± 2
6	Thujone	17.103	1107	1103	24 ± 1
7	β-Thujone	17.804	1121	1114	4.8 ± 0.3
8	Camphor	18.994	1146	1142	29 ± 1
9	Isoborneol	20.079	1168	1157	5.0 ± 0.4
10	α-Terpineol	21.129	1190	1189	0.46 ± 0.04
11	Bornyl acetate	25.102	1276	1284	0.8 ± 0.1
12	β-Caryophyllene	30.72	1404	1419	0.466 ± 0.002
13	Humulene	32.225	1441	1454	0.69 ± 0.03
	Total identified (%)				91.24 ± 0.05
	Monoterpenes				9.02 ± 0.66
	Oxygenated monoterpenes				81 ± 5
	Sesquiterpenes				1.16 ± 0.03
	Not identified				8.76 ± 0.14
*Thymus vulgaris* L.					
Number	Compound	RT (min)	LRI ^a^	LRI ^b^	Relative % ^c^
1	α-Pinene	8.475	926	932	0.012 ± 0.001
2	Camphene	9.14	940	946	0.005 ± 0.0002
3	β-Pinene	11.24	985	974	0.17 ± 0.01
4	*o*-Cymene	13.13	1026	1022	25.324 ± 0.04
5	ρ-Cymene	13.218	1028	1023	14 ± 1
6	Limonene	13.28	1029	1024	0.18 ± 0.01
7	Eucalyptol	13.341	1030	1031	0.648 ± 0.03
8	γ-Terpinene	14.461	1052	1054	0.278 ± 0.002
9	Camphor	18.591	1138	1141	0.26 ± 0.004
10	Thymol methyl ether	22.739	1223	12,132	0.609 ± 0.01
11	Methyl carvacrol	23.142	1231	1241	0.54 ± 0.01
12	Thymol	26.31	1296	1289	41 ± 1
13	Carvacrol	26.52	1300	1298	5.1 ± 0.2
14	γ-Muurolene	34.676	1496	1478	0.1 ± 0.01
15	Caryophyllene oxide	37.371	1558	1582	1.4 ± 0.1
16	δ-Cadinol	39.769	1613	1638	0.213 ± 0.01
	Total identified (%)				90.3 ± 0.4
	Monoterpenes				39.7 ± 1.1
	Oxygenated monoterpenes				47.74 ± 0.52
	Sesquiterpenes				0.10 ± 0.01
	Oxygenated sesquiterpenes				1.61 ± 0.11
	Others (%)				1.15 ± 0.02
	Not identified				9.7 ± 0.4

^a^ LRI, linear retention index determined on a DB-5MS fused-silica column relative to a series of *n*-alkanes (C8–C40). ^b^ Linear retention index reported in the literature (Adams, 2017). ^c^ Relative % is given as the mean ± SD (*n* = 3).

**Table 2:** Antioxidant activity (reducing power and DPPH assays) of Lamiaceae essential oils (EC_50_ (mg/mL)).

	Antioxidant Activity	
	RP	DPPH
*O. vulgare*	1.69 ± 0.07 ^c^	9.2 ± 0.6 ^d^
*R. officinalis*	2.79 ± 0.02 ^b^	55.9 ± 0.5 ^a^
*S. officinalis*	6.50 ± 0.23 ^a^	39.92 ± 1.21 ^b^
*T. vulgaris*	1.63 ± 0.04 ^c^	10.68 ± 0.31 ^c^
E223	0.053 ± 0.002	0.043 ± 0.004
E302	0.020 ± 0.003	0.009 ± 0.001
BHT	0.045 ± 0.001	0.071 ± 0.004

BHT, butylated hydroxytoluene; E302, calcium ascorbate; and E223, sodium metabisulphite. In each column, different letters (a, b, c and d) mean significant differences (*p* < 0.05) between extracts.

**Table 3:** Cytotoxicity activity of Lamiaceae essential oils (GI_50_ (µg/mL)).

	Tumoral Cell Lines (GI_50_ Values; µg/mL)				Non-Tumoral Culture (GI_50_ Values; µg/mL)
	AGS	CaCo-2	MCF7	NCI-H460	VERO
*O. vulgare*	48 ± 4 ^c^	45 ± 4 ^c^	45 ± 4 ^d^	84 ± 3 ^c^	>400 ^a^
*R. officinalis*	60 ± 3 ^c^	221 ± 11 ^a^	202 ± 14 ^b^	306 ± 11 ^a^	>400 ^a^
*S. officinalis*	236 ± 14 ^a^	147 ± 16 ^b^	249 ± 21 ^a^	305 ± 19 ^a^	243 ± 21 ^b^
*T. vulgaris*	175 ± 11 ^b^	156 ± 10 ^b^	159 ± 13 ^c^	243 ± 16 ^b^	243 ± 11 ^b^
Ellipticine (µM)	0.9 ± 0.1	0.8 ± 0.1	1.020 ± 0.004	1.01 ± 0.01	0.6 ± 0.1

AGS, human gastric epithelial cell line; CaCo2, human colorectal adenocarcinoma cell line; MCF7, human breast carcinoma cell line; NCI-H460, human lung carcinoma cell line; and VERO, African green monkey kidney cell line. In each column, different letters (a, b, c and d) mean significant differences (*p* < 0.05) between extracts.

**Table 4:** Anti-inflammatory activity of Lamiaceae essential oils (GI_50_ (µg/mL)).

	Anti-Inflammatory
	(GI_50_ Values; µg/mL)
	RAW264.7
*O. vulgare*	13.3 ± 0.5 ^b^
*R. officinalis*	58.1 ± 1 ^a^
*S. officinalis*	9.5 ± 0.1 ^c^
*T. vulgaris*	8 ± 1 ^d^
Dexametasone (µM)	16 ± 1

In each column, different letters (a, b, c and d) mean significant differences (*p* < 0.05) between extracts.

**Table 5:** Antibacterial and antifungal activities of Lamiaceae essential oils (% (*v*/*v*)).

	Antibacterial Activity													
	*O. vulgare*		*T. vulgaris*		*S. officinalis*		*R. officinalis*		Positive Control					
									Streptomycin 1 mg/mL		Methicillin 1 mg/mL		Ampicillin 10 mg/mL	
	MIC	MBC	MIC	MBC	MIC	MBC	MIC	MBC	MIC	MBC	MIC	MBC	MIC	MBC
	Gram-negative bacteria													
*Escherichia coli*	0.1	0.08	0.3	0.31	2.5	2.5	2.5	2.5	0.01	0.01	n.t.	n.t.	0.2	0.15
*Pseudomonas aeruginosa*	2.5	2.5	>2.5	2.5	>2.5	2.5	>2.5	2.5	0.06	0.06	n.t.	n.t.	0.6	0.63
*Salmonella enterica*	0.2	0.16	0.6	1.25	2.5	2.5	2.5	2.5	0.01	0.01	n.t.	n.t.	0.2	0.15
*Yersinia enterocolitica*	0.2	0.3	0.3	0.6	2.5	2.5	2.5	2.5	0.01	0.01	n.t.	n.t.	0.2	0.15
	Gram-positive bacteria													
*Bacillus cereus*	0.2	2.5	0.6	2.5	2.5	2.5	2.5	2.5	0.01	0.01	n.t.	n.t.	n.t.	n.t.
*Listeria monocytogenes*	0.1	2.5	0.6	2.5	1.25	2.5	2.5	2.5	0.01	0.01	n.t.	n.t.	0.2	0.15
*Staphylococcus aureus*	0.3	0.63	1.3	2.5	2.5	2.5	2.5	2.5	0.01	0.01	0.01	0.01	0.2	0.15
	Antifungal Activity													
											Ketaconazole 1 mg/mL			
	MIC	MFC	MIC	MFC	MIC	MFC	MIC	MFC			MIC	MFC		
*Aspergillus brasiliensis*	0.1	0.31	0.3	0.31	0.31	0.63	0.31	0.63			0.06	0.13		
*Aspergillus fumigatus*	0.1	0.31	0.1	0.31	0.08	0.31	0.08	0.31			0.5	1		

Essential oils were tested in the concentration range of 2.5% to 0.039% (*v*/*v*). MIC, minimum inhibitory concentration; MBC, minimum bactericidal concentration; MFC, minimum fungicidal concentration. n.t.—non tested.

## Data Availability

The original contributions presented in the study are included in the article/Supplementary Material; further inquiries can be directed to the corresponding author/s.

## Supplementary Materials

The following supporting information can be downloaded at https://www.mdpi.com/article/10.3390/molecules29122827/s1: Chromatograms obtained by GC/MS of essential oils. Extraction methodology and extraction parameters are described in Section 3.2.

## References

[1] Karpiński T.M. (2020). Essential Oils of Lamiaceae Family Plants as Antifungals. Biomolecules.

[2] Kozłowska M., Laudy A.E., Przybył J., Ziarno M., Majewska E. (2015). Chemical composition and antibacterial activity of some medicinal plants from lamiaceae family. Acta Pol. Pharm..

[3] Ramos da Silva L.R., Ferreira O.O., Cruz J.N., de Jesus Pereira Franco C., Oliveira dos Anjos T., Cascaes M.M., Almeida da Costa W., Helena de Aguiar Andrade E., Santana de Oliveira M. (2021). Lamiaceae Essential Oils, Phytochemical Profile, Antioxidant, and Biological Activities. Evid.-Based Complement. Altern. Med..

[4] Stefanaki A., van Andel T. (2021). Mediterranean Aromatic Herbs and Their Culinary Use. Aromatic Herbs in Food.

[5] Xavier V., Spréa R., Finimundy T.C., Heleno S.A., Amaral J.S., Barros L., Ferreira I.C.F.R., Carocho M., Heleno S.A., Barros L. (2023). Terpenes. Natural Secondary Metabolites: From Nature, through Science, to Industry.

[6] Jackson-Davis A., White S., Kassama L.S., Coleman S., Shaw A., Mendonca A., Cooper B., Thomas-Popo E., Gordon K., London L. (2023). A Review of Regulatory Standards and Advances in Essential Oils as Antimicrobials in Foods. J. Food Prot..

[7] Ju J., Chen X., Xie Y., Yu H., Guo Y., Cheng Y., Qian H., Yao W. (2019). Application of Essential Oil as a Sustained Release Preparation in Food Packaging. Trends Food Sci. Technol..

[8] Sharma S., Cheng S.-F., Bhattacharya B., Chakkaravarthi S. (2019). Efficacy of Free and Encapsulated Natural Antioxidants in Oxidative Stability of Edible Oil: Special Emphasis on Nanoemulsion-Based Encapsulation. Trends Food Sci. Technol..

[9] Sharma S., Mulrey L., Byrne M., Jaiswal A.K., Jaiswal S. (2022). Encapsulation of Essential Oils in Nanocarriers for Active Food Packaging. Foods.

[10] Garzoli S., Petralito S., Ovidi E., Turchetti G., Laghezza Masci V., Tiezzi A., Trilli J., Cesa S., Casadei M.A., Giacomello P. (2020). Lavandula x Intermedia Essential Oil and Hydrolate: Evaluation of Chemical Composition and Antibacterial Activity before and after Formulation in Nanoemulsion. Ind. Crops Prod..

[11] Seow Y.X., Yeo C.R., Chung H.L., Yuk H.-G. (2014). Plant Essential Oils as Active Antimicrobial Agents. Crit. Rev. Food Sci. Nutr..

[12] Hyldgaard M., Mygind T., Meyer R.L. (2012). Essential Oils in Food Preservation: Mode of Action, Synergies, and Interactions with Food Matrix Components. Front. Microbiol..

[13] Diass K., Brahmi F., Mokhtari O., Abdellaoui S., Hammouti B. (2021). Biological and Pharmaceutical Properties of Essential Oils of *Rosmarinus officinalis* L. and *Lavandula officinalis* L.. Mater. Today Proc..

[14] Guzmán E., Lucia A. (2021). Essential Oils and Their Individual Components in Cosmetic Products. Cosmetics.

[15] Ebadollahi A., Ziaee M., Palla F. (2020). Essential Oils Extracted from Different Species of the Lamiaceae Plant Family as Prospective Bioagents against Several Detrimental Pests. Molecules.

[16] Mączka W., Twardawska M., Grabarczyk M., Wińska K. (2023). Carvacrol—A Natural Phenolic Compound with Antimicrobial Properties. Antibiotics.

[17] Caputo L., Amato G., de Bartolomeis P., De Martino L., Manna F., Nazzaro F., De Feo V., Barba A.A. (2022). Impact of Drying Methods on the Yield and Chemistry of *Origanum vulgare* L. Essential Oil. Sci. Rep..

[18] Elshafie H., Armentano M., Carmosino M., Bufo S., De Feo V., Camele I. (2017). Cytotoxic Activity of *Origanum vulgare* L. on Hepatocellular Carcinoma Cell Line HepG2 and Evaluation of Its Biological Activity. Molecules.

[19] Rathod N.B., Kulawik P., Ozogul F., Regenstein J.M., Ozogul Y. (2021). Biological Activity of Plant-Based Carvacrol and Thymol and Their Impact on Human Health and Food Quality. Trends Food Sci. Technol..

[20] Mauriello E., Ferrari G., Donsì F. (2021). Effect of Formulation on Properties, Stability, Carvacrol Release and Antimicrobial Activity of Carvacrol Emulsions. Colloids Surf. B Biointerfaces.

[21] Rúa J., del Valle P., de Arriaga D., Fernández-Álvarez L., García-Armesto M.R. (2019). Combination of Carvacrol and Thymol: Antimicrobial Activity Against *Staphylococcus Aureus* and Antioxidant Activity. Foodborne Pathog. Dis..

[22] Günes-Bayir A., Kiziltan H.S., Kocyigit A., Güler E.M., Karataş E., Toprak A. (2017). Effects of Natural Phenolic Compound Carvacrol on the Human Gastric Adenocarcinoma (AGS) Cells in Vitro. Anti-Cancer Drugs.

[23] Ed-Dra A., Nalbone L., Filali F.R., Trabelsi N., El Majdoub Y.O., Bouchrif B., Giarratana F., Giuffrida A. (2021). Comprehensive Evaluation on the Use of *Thymus vulgaris* Essential Oil as Natural Additive against Different Serotypes of Salmonella Enterica. Sustainability.

[24] Galovičová L., Borotová P., Valková V., Vukovic N.L., Vukic M., Štefániková J., Ďúranová H., Kowalczewski P.Ł., Čmiková N., Kačániová M. (2021). *Thymus vulgaris* Essential Oil and Its Biological Activity. Plants.

[25] Marchese A., Orhan I.E., Daglia M., Barbieri R., Di Lorenzo A., Nabavi S.F., Gortzi O., Izadi M., Nabavi S.M. (2016). Antibacterial and Antifungal Activities of Thymol: A Brief Review of the Literature. Food Chem..

[26] Escobar A., Pérez M., Romanelli G., Blustein G. (2020). Thymol Bioactivity: A Review Focusing on Practical Applications. Arab. J. Chem..

[27] Nikolić M., Glamočlija J., Ferreira I.C.F.R., Calhelha R.C., Fernandes Â., Marković T., Marković D., Giweli A., Soković M. (2014). Chemical Composition, Antimicrobial, Antioxidant and Antitumor Activity of *Thymus serpyllum* L., Thymus Algeriensis Boiss. and Reut and *Thymus vulgaris* L. Essential Oils. Ind. Crops Prod..

[28] de Oliveira J.R., de Jesus Viegas D., Martins A.P.R., Carvalho C.A.T., Soares C.P., Camargo S.E.A., Jorge A.O.C., de Oliveira L.D. (2017). *Thymus vulgaris* L. Extract Has Antimicrobial and Anti-Inflammatory Effects in the Absence of Cytotoxicity and Genotoxicity. Arch. Oral Biol..

[29] Costa M.F., Durço A.O., Rabelo T.K., Barreto R.d.S., Guimarães A.G. (2019). Effects of Carvacrol, Thymol and Essential Oils Containing Such Monoterpenes on Wound Healing: A Systematic Review. J. Pharm. Pharmacol..

[30] Milovanovic S., Markovic D., Mrakovic A., Kuska R., Zizovic I., Frerich S., Ivanovic J. (2019). Supercritical CO_2_—Assisted Production of PLA and PLGA Foams for Controlled Thymol Release. Mater. Sci. Eng. C.

[31] Spisni E., Petrocelli G., Imbesi V., Spigarelli R., Azzinnari D., Donati Sarti M., Campieri M., Valerii M.C. (2020). Antioxidant, Anti-Inflammatory, and Microbial-Modulating Activities of Essential Oils: Implications in Colonic Pathophysiology. Int. J. Mol. Sci..

[32] Amina B., Soumeya B., Salim B., Mahieddine B., Sakina B., Chawki B., Francesca N., Marzia V., Carmine N., Luigi D.B. (2022). Chemical Profiling, Antioxidant, Enzyme Inhibitory and in Silico Modeling of *Rosmarinus officinalis* L. and Artemisia Herba Alba Asso. Essential Oils from Algeria. S. Afr. J. Bot..

[33] Yang P., Jia M., Zhu L. (2021). Acaricidal Activity of the Essential Oil from Senecio Cannabifolius and Its Constituents Eucalyptol and Camphor on Engorged Females and Larvae of Rhipicephalus Microplus (Acari: Ixodidae). Exp. Appl. Acarol..

[34] Kim T., Song B., Cho K.S., Lee I.-S. (2020). Therapeutic Potential of Volatile Terpenes and Terpenoids from Forests for Inflammatory Diseases. Int. J. Mol. Sci..

[35] Ağuş H., Yilmaz S., Şengoz C. (2019). Crosstalk between Autophagy and Apoptosis Induced by Camphor inSchizosaccharomyces Pombe. Turk. J. Biol..

[36] El Euch S.K., Hassine D.B., Cazaux S., Bouzouita N., Bouajila J. (2019). Salvia Officinalis Essential Oil: Chemical Analysis and Evaluation of Anti-Enzymatic and Antioxidant Bioactivities. S. Afr. J. Bot..

[37] Khedher M.R.B., Khedher S.B., Chaieb I., Tounsi S., Hammami M. (2017). Chemical Composition and Biological Activities of Salvia Officinalis Essential Oil from Tunisia. EXCLI J..

[38] Laothaweerungsawat N., Sirithunyalug J., Chaiyana W. (2020). Chemical Compositions and Anti-Skin-Ageing Activities of *Origanum vulgare* L. Essential Oil from Tropical and Mediterranean Region. Molecules.

[39] Mancini E., Senatore F., Del Monte D., De Martino L., Grulova D., Scognamiglio M., Snoussi M., De Feo V. (2015). Studies on Chemical Composition, Antimicrobial and Antioxidant Activities of Five *Thymus vulgaris* L. Essential Oils. Molecules.

[40] El Kharraf S., El-Guendouz S., Farah A., Bennani B., Mateus M.C., El Hadrami E.M., Miguel M.G. (2021). Hydrodistillation and Simultaneous Hydrodistillation-Steam Distillation of *Rosmarinus officinalis* and Origanum Compactum: Antioxidant, Anti-Inflammatory, and Antibacterial Effect of the Essential Oils. Ind. Crops Prod..

[41] Oualdi I., Diass K., Azizi S., Dalli M., Touzani R., Gseyra N., Yousfi E.B. (2022). *Rosmarinus officinalis* Essential Oils from Morocco: New Advances on Extraction, GC/MS Analysis, and Antioxidant Activity. Nat. Prod. Res..

[42] Hlwatika C.N.M., Bhat R.B. (2002). An Ecological Interpretation of the Difference in Leaf Anatomy and Its Plasticity in Contrasting Tree Species in Orange Kloof, Table Mountain, South Africa. Ann. Bot..

[43] Gonzalez-Burgos E., Gomez-Serranillos M.P. (2012). Terpene Compounds in Nature: A Review of Their Potential Antioxidant Activity. CMC.

[44] Han X., Parker T.L. (2017). Anti-Inflammatory, Tissue Remodeling, Immunomodulatory, and Anticancer Activities of Oregano (*Origanum vulgare*) Essential Oil in a Human Skin Disease Model. Biochim. Open.

[45] Dolghi A., Coricovac D., Dinu S., Pinzaru I., Dehelean C.A., Grosu C., Chioran D., Merghes P.E., Sarau C.A. (2022). Chemical and Antimicrobial Characterization of *Mentha Piperita* L. and *Rosmarinus officinalis* L. Essential Oils and In Vitro Potential Cytotoxic Effect in Human Colorectal Carcinoma Cells. Molecules.

[46] Abdelli W., Bahri F., Romane A., Höferl M., Wanner J., Schmidt E., Jirovetz L. (2017). Chemical Composition and Anti-Inflammatory Activity of Algerian *Thymus vulgaris* Essential Oil. Nat. Prod. Commun..

[47] Carbone C., Martins-Gomes C., Caddeo C., Silva A.M., Musumeci T., Pignatello R., Puglisi G., Souto E.B. (2018). Mediterranean Essential Oils as Precious Matrix Components and Active Ingredients of Lipid Nanoparticles. Int. J. Pharm..

[48] Mohammed H.A., Eldeeb H.M., Khan R.A., Al-Omar M.S., Mohammed S.A.A., Sajid M.S.M., Aly M.S.A., Ahmad A.M., Abdellatif A.A.H., Eid S.Y. (2021). Sage, Salvia Officinalis L., Constituents, Hepatoprotective Activity, and Cytotoxicity Evaluations of the Essential Oils Obtained from Fresh and Differently Timed Dried Herbs: A Comparative Analysis. Molecules.

[49] Tosun A., Khan S., Kim Y., Calín-Sánchez A., Hysenaj X., Carbonell-Barrachina A. (2014). Essential Oil Composition and Anti-Inflammatory Activity of *Salvia officinalis* L (Lamiaceae) in Murin Macrophages. Trop. J. Pharm. Res..

[50] Simirgiotis M.J., Burton D., Parra F., López J., Muñoz P., Escobar H., Parra C. (2020). Antioxidant and Antibacterial Capacities of *Origanum vulgare* L. Essential Oil from the Arid Andean Region of Chile and Its Chemical Characterization by GC-MS. Metabolites.

[51] Kosakowska O., Węglarz Z., Pióro-Jabrucka E., Przybył J.L., Kraśniewska K., Gniewosz M., Bączek K. (2021). Antioxidant and Antibacterial Activity of Essential Oils and Hydroethanolic Extracts of Greek Oregano (*O. vulgare* L. Subsp. Hirtum (Link) Ietswaart) and Common Oregano (*O. vulgare* L. Subsp. Vulgare). Molecules.

[52] Borugă O., Jianu C., Mişcă C., Goleţ I., Gruia A., Horhat F. (2014). *Thymus vulgaris* Essential Oil: Chemical Composition and Antimicrobial Activity. J. Med. Life.

[53] De Martino L., De Feo V., Nazzaro F. (2009). Chemical Composition and in Vitro Antimicrobial and Mutagenic Activities of Seven Lamiaceae Essential Oils. Molecules.

[54] Nieto G. (2017). Biological Activities of Three Essential Oils of the Lamiaceae Family. Medicines.

[55] Waller S.B., Cleff M.B., Serra E.F., Silva A.L., Gomes A.d.R., de Mello J.R.B., de Faria R.O., Meireles M.C.A. (2017). Plants from Lamiaceae Family as Source of Antifungal Molecules in Humane and Veterinary Medicine. Microb. Pathog..

[56] Sprea R.M., Fernandes L.H.M., Pires T.C.S.P., Calhelha R.C., Rodrigues P.J., Amaral J.S. (2023). Volatile Compounds and Biological Activity of the Essential Oil of Aloysia Citrodora Paláu: Comparison of Hydrodistillation and Microwave-Assisted Hydrodistillation. Molecules.

[57] Xavier V., Finimundy T.C., Heleno S.A., Amaral J.S., Calhelha R.C., Vaz J., Pires T.C.S.P., Mediavilla I., Esteban L.S., Ferreira I.C.F.R. (2021). Chemical and Bioactive Characterization of the Essential Oils Obtained from Three Mediterranean Plants. Molecules.

