# Supplementary material for: Chemical and Bioactive Evaluation of Essential Oils from Edible and Aromatic Mediterranean Lamiaceae Plants
Source: Molecules. 2024 Jun 13;29(12):2827. doi: 10.3390/molecules29122827 (PMC11206263; doi:10.3390/molecules29122827)

Supplementary Materials:

Chromatograms obtained by GC/MS of essential oils. Extraction methodology as well as extraction parameters are described in Section 3.2.

Rosmarinus officinalis L.

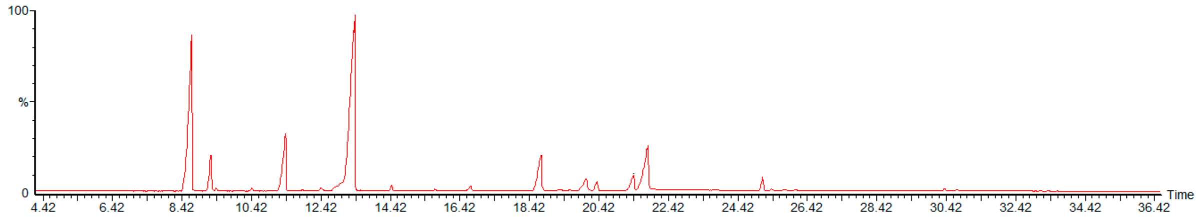

Salvia officinallis L.

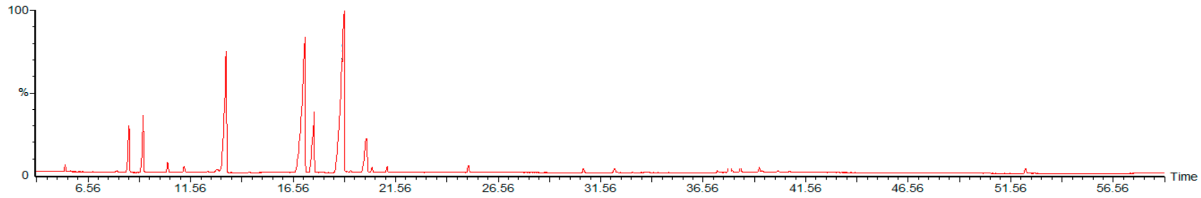

Origanum vulgare L.

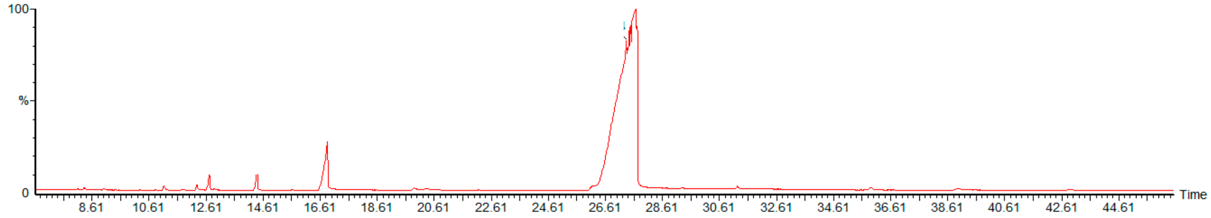

Thymus vulgaris L.

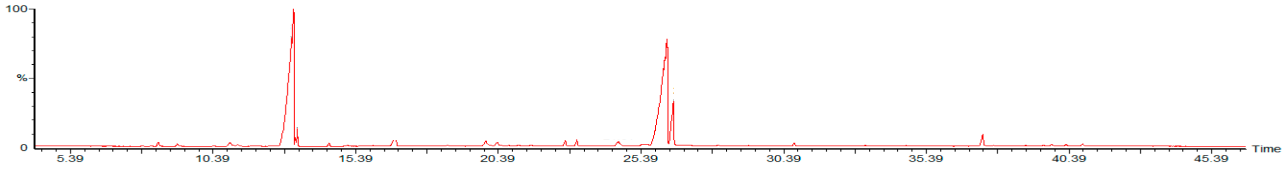

Supplement: Supplementary file 1 [file molecules-29-02827-s001.zip › molecules-3015597-supplementary.pdf]
